# A Rare Case of Ecchordosis Physaliphora Presenting With Headache, Abducens Nerve Palsy, and Intracranial Hypertension

**DOI:** 10.7759/cureus.8843

**Published:** 2020-06-26

**Authors:** Ruiqing Sun, Yousaf Ajam, Gerald Campbell, Todd Masel

**Affiliations:** 1 Neurology, University of Texas Medical Branch, Galveston, USA; 2 Pathology, University of Texas Medical Branch, Galveston, USA

**Keywords:** ecchordosis physaliphora, headache, abducen nerve palsy, intracranial hypertension

## Abstract

We report a rare case of ecchordosis physaliphora presenting with headache, nausea, and diplopia. On neurological examination, the patient was found to have left abducens nerve palsy. CT of the head without contrast was unremarkable. Brain MRI demonstrated a non-enhancing retroclival mass with a mass effect upon the ventral pons. The mass had increased signal intensity on T2 and decreased signal intensity on T1-weighted sequences. Lumbar puncture revealed an opening pressure of 37 cm H_2_O. The patient underwent an endoscopic endonasal approach for retroclival mass resection three weeks later. The tissue analysis of the mass was consistent with ecchordosis physaliphora. This could have been misdiagnosed as idiopathic intracranial hypertension had the MRI of the brain not been performed.

## Introduction

Ecchordosis physaliphora is a congenital benign hamartomatous lesion derived from notochord remnants. It is usually located in the retroclival prepontine region but can be found anywhere from the skull base to the sacrum [1]. Unlike chordomas, which are often symptomatic due to brainstem or cranial nerve compression, patients with ecchordosis physaliphora are usually asymptomatic. They are found in 0.5-2% of autopsies [2]. However, there are several case reports describing symptomatic ecchordosis physaliphora as well. The most common symptoms include headache, pneumocephalus, spontaneous cerebrospinal fluid (CSF) rhinorrhea with meningitis, and abducens nerve palsy [3-7]. The differential diagnoses of retroclival intradural lesions consist mainly of chordoma, ecchordosis physaliphora, skull base metastasis, dermoid cyst, epidermoid cyst, and arachnoid cyst. Distinguishing ecchordosis physaliphora from its malignant counterpart, chordoma, is very important because of the aggressive nature of the chordoma. CT is generally not sensitive for such lesions, primarily due to posterior fossa artifacts and the near-CSF density of the mass. The current gold-standard test for the diagnosis of ecchordosis physaliphora is brain MRI with and without contrast as well as pathology testing [1,8,9]. It is worth mentioning, however, that ecchordosis physaliphora and chordoma are histologically indistinguishable in small fragmentary specimens, and are generally differentiated by examining the margins, with the latter demonstrating infiltrative growth [1].

## Case presentation

**Figure 1:**
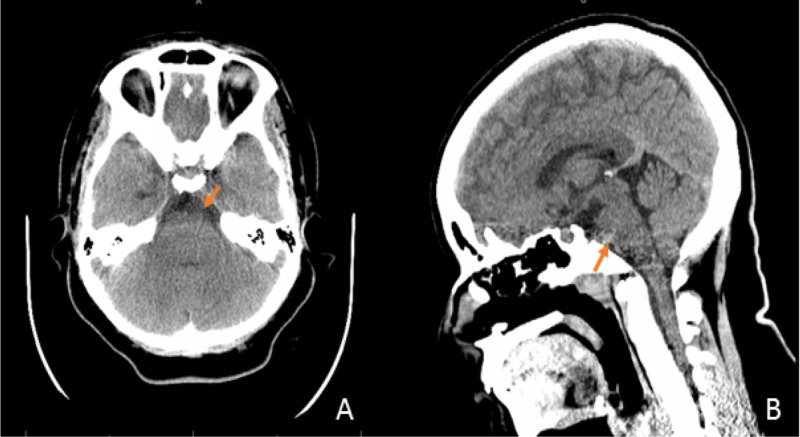
CT head without contrast at admission CT head without contrast performed at admission: an isodense retroclival mass with mild mass effect on the pons (arrow). A: axial view; B: sagittal view. This was read as unremarkable before the brain MRI was performed CT: computed tomography; MRI: magnetic resonance imaging

**Figure 2:**
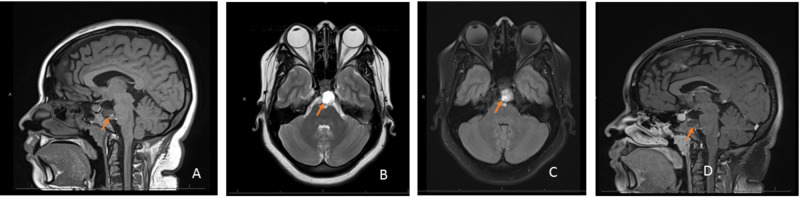
Brain MRI with and without contrast Brain MRI with and without contrast performed on admission. A - sagittal T1-weighted view: the mass appears heterogeneously hypointense (arrow); B - axial T2-weighted view: the mass appears homogenously hyperintense (arrow); C - axial FLAIR: the mass has slightly heterogeneously hyperintense diffusion (arrow); D - gadolinium-enhanced sagittal T1-weighted view: a lesion in the retroclival prepontine location with no contrast enhancement (arrow) MRI: magnetic resonance imaging; FLAIR: fluid attenuation inversion recovery

Our patient was a 22-year-old female with a past medical history of headache who was admitted for severe headache, nausea, and diplopia. One day prior to presentation, the patient had awoken with a severe headache. She described her headache as located in the occipital region as well as the left frontal region and around the left eye. The associated symptoms were nausea, vomiting, and blurred vision, and there was no photophobia or phonophobia. The headache was aggravated by head movement, lying down, coughing, and bending over, and it was slightly alleviated by ibuprofen. The next day, when she awoke in the morning, she had blurred vision and binocular horizontal diplopia. Of note, the patient had been experiencing intermittent occipital headaches for almost one year, slightly relieved by over-the-counter oral analgesics [acetaminophen, nonsteroidal anti-inflammatory drugs (NSAIDS)]. She also reported a 30-lbs unintentional weight-gain over the past year. Neurological exam revealed impaired left eye abduction without papilledema or other neurological deficits. CT head without contrast was unremarkable (Figure 1A, 1B). In order to rule out subarachnoid hemorrhage, a lumbar puncture was performed, which showed an opening pressure of 37 cm H_2_O; 28 CC of CSF was removed, and the subsequent closing pressure was 22 cm H_2_O. The CSF examination was unremarkable (Table 1). Finally, brain MRI with and without contrast was performed and showed a non-enhancing retroclival mass with mass effect upon the ventral pons, which had increased signal intensity on T2 and decreased signal intensity on T1-weighted sequences, and was highly suspicious for ecchordosis physaliphora (Figure 2A, 2B, 2C, 2D). Although the patient’s headache had improved after the lumbar puncture, the diplopia persisted. The patient developed a post-lumbar puncture headache on the second day, and this resolved after intravenous hydration. She was discharged with a prescription for acetazolamide 500 mg twice a day.

**Figure 3:**
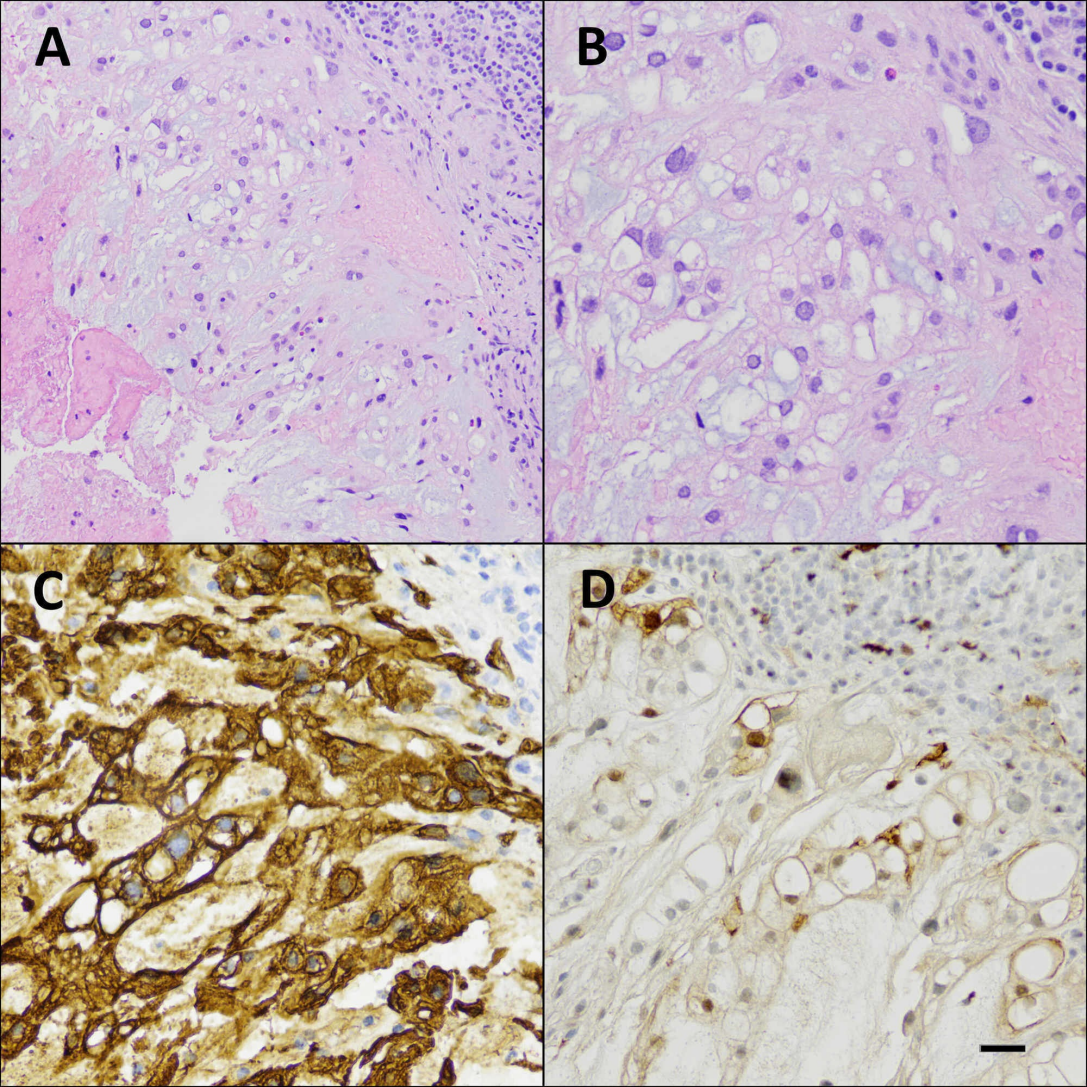
Histopathology of the retroclival mass Low-magnification (A) and high-magnification (B) images of hematoxylin and eosin-stained, formalin-fixed, paraffin-embedded sections of the lesion, showing physaliphorous cells (center) and lymphocytic infiltrates in adjacent tissue included in the biopsy (upper right). Immunohistochemical stains for pancytokeratins (C) and S100 (D), showing strong reactivity of tumor cells for cytokeratins (AE1/AE3) and weak reactivity for S100. Scale bar (lower right) represents 50 microns for Figure 3A and 25 microns for Figures 3B-3D. Note that the distinction between chordoma and ecchordosis physaliphora is not readily determined solely from this histology but is dependent on biologic behavior including invasiveness

Three weeks later, the patient underwent an endoscopic endonasal approach for retroclival mass resection. The hematoxylin and eosin-stained, formalin-fixed, paraffin-embedded sections of the lesion showed physaliphorous cells and lymphocytic infiltrates in adjacent tissue; immunohistochemical stains showed strong reactivity of tumor cells for cytokeratin (AE1/AE3) and weak reactivity for S100. The surgical finding and tissue analysis of the mass were consistent with ecchordosis physaliphora (Figure 3). After surgery, at the two-month follow-up, her headache had resolved, but she had persistent diplopia; at five months, diplopia was found resolved and the patient was taken off Diamox (Teva Pharmaceuticals, Parsippany, NJ); at eight months, the patient had no symptoms and a repeated brain MRI showed no tumor recurrence.

**Table 1:** CSF test results CSF: cerebrospinal fluid; WBC: white blood cells; RBC: red blood cells

CSF test	Result
Cell count	WBC 1, RBC 0
Chemistry	Glucose 52, protein 38
Meningitis/encephalitis panel	Negative
Electrophoresis	Negative
Aquaporin-4	Negative
Cytology	No malignant cells

## Discussion

Ecchordosis physaliphora is a congenital benign hamartomatous lesion. It is usually asymptomatic and incidentally found in 0.5-2% of autopsies. Our patient presented with an acute severe headache and sudden-onset diplopia. Lumbar puncture demonstrated intracranial hypertension with an opening pressure of 37 cm H_2_O. Given the brain MRI findings and follow-up outcomes, we believe that direct compression from the tumor and increased intracranial pressure contributed to the patient’s symptoms.

The diagnosis of ecchordosis physaliphora can be challenging without the aid of an MRI. The initial head CT was read as normal in this case. In cases of intracranial hypertension and obesity, idiopathic intracranial hypertension is often atop the list of differential diagnoses. In this case, however, there was no papilledema on the fundoscopic exam. Brain MRI with and without contrast revealed the possible diagnosis of ecchordosis physaliphora with specific features: non-enhancing retroclival mass with increased signal intensity on T2-weighted sequences and decreased signal intensity on T1-weighted sequences [1,8,9]. The malignant counterpart, chordoma, usually presents as an enhancing retroclival mass with hyperintensity on both T1 and T2-weighted sequences, due to intra-tumoral calcification, increased vascularity, and disruption of the blood-brain barrier. Furthermore, the tissue analysis confirmed the diagnosis of ecchordosis physaliphora. 

No previous case reports regarding ecchordosis physaliphora have mentioned raised opening pressure in the lumbar puncture. Our patient's initial brain MRI showed non-enhancing retroclival mass with mass effect upon the ventral pons and the left sixth cranial nerve stretch. The mass with a size of 17 mm x 15 mm x 19 mm at the prepontine region might cause intracranial hypertension even though brain MRI did not show any radiographic features for increased intracranial pressure. While the patient did not have papilledema, we could not rule out idiopathic intracranial hypertension completely due to the patient’s history, which was the reason why the patient was placed on Diamox initially. 

A lesson learned from this case is that a brain MRI with and without contrast is essential for the diagnosis of ecchordosis physaliphora and that this diagnosis should be considered in cases that show similarity to idiopathic intracranial hypertension. In our patient, ecchordosis physaliphora could have been misdiagnosed as idiopathic intracranial hypertension had the MRI of the brain not been performed.

## Conclusions

In this report, we discussed a rare case of symptomatic ecchordosis physaliphora presenting with headache, nausea, abducens nerve palsy, and intracranial hypertension. The diagnosis was revealed by brain MRI with and without contrast as well as tissue analysis.
